# Advances in Polyimide Membranes for Gas Separation: Synthesis, Modification, and Application

**DOI:** 10.3390/molecules30173507

**Published:** 2025-08-27

**Authors:** Qiu-Ying Zhang, Heng Mao, Meng Wen, Bing-Hong Chen, Qian-Qian Li, Yan-Mei Zhang, Zhi-Ping Zhao

**Affiliations:** School of Chemistry and Chemical Engineering, Beijing Institute of Technology, Beijing 102488, China; qiuyinglucky@163.com (Q.-Y.Z.); 3120225638@bit.edu.cn (M.W.); c3bh17@163.com (B.-H.C.); li014104@163.com (Q.-Q.L.); xiaomei202205@163.com (Y.-M.Z.)

**Keywords:** polyimide, synthesis approach, membrane preparation, modification, gas separation

## Abstract

Membrane technology is widely used in gas separation processes due to its small footprint, high energy efficiency, and favorable economic viability. The current membrane market predominantly relies on polymer membranes, among which polyimide (PI) membranes stand out as highly promising materials due to their superior gas separation performance coupled with exceptional thermal and chemical stability. However, traditional polyimide membranes suffer from low gas permeability and insufficient plasticization resistance, hindering their broader industrial application. In order to meet the demands of more stringent application fields, it is crucial to further improve their gas performance and anti-plasticization to enhance their cost-effectiveness. Consequently, it is essential to modify traditional polyimides and formulate membrane fabrication strategies to solve these problems. This review introduces the monomer structures and synthesis approaches of polyimides, including solution-based and solid-state thermal condensation. Then, we propose representative preparation methods of polyimide-based membranes. Additionally, modification strategies, including thermal rearrangement, cross-linking, and physical blending, are summarized, which address the critical issues in contemporary polyimide-based gas separation membranes. Finally, this review critically discusses the current challenges and prospects for developing polyimide membranes for gas separation.

## 1. Introduction

With continuous economic development and the intensification of the energy crisis, the research and application of gas separation technology have attracted widespread attention. Compared with the conventional separation technologies (e.g., distillation and absorption), membrane-based gas separation technology operates without phase change, which offers distinct advantages such as high efficiency, low energy consumption, environmental sustainability, and industrial scalability [1,2,3]. Since the 1970s, the gas separation membrane market has demonstrated a remarkable upward trend and is expected to continue growing with advancing technology and expanding applications [4].

Membrane materials are the critical determinant for gas separation membrane technology. Polymer membranes exhibit exceptional processability and economic viability, rendering them attractive options for large-scale gas separation applications [5,6]. To date, common polymer membrane materials, such as polysulfone (PSf), cellulose acetate (CA), and polyimide (PI), have been widely utilized in natural gas purification, hydrogen recovery, and olefin/paraffin separation [7,8,9,10,11]. Among them, PI, a polymer material containing an imide heterocyclic ring, has been a major research focus for molecular separation due to its outstanding thermal stability, chemical resistance, and mechanical strength. Aromatic polyimides can be adjusted by introducing different types of dianhydride and diamine monomers, thereby modifying the free volume and rigidity of the polyimide matrix, which significantly affects its gas permeability and selectivity. Xing et al. [12] developed fluorinated polyimide membranes for the efficient separation and enrichment of helium from natural gas. The optimized membrane demonstrated a helium permeability of 73 Barrer and helium/methane selectivity of 247. Over the past two decades, polyimide membranes have gained increasing attention for gas separation, showing a consistent growth trend. In the 1960s, DuPont pioneered the commercialization of PI membranes [10]. Typically, aromatic dianhydride and diamine were used in low-temperature condensation polymerization to produce polyamide acid, which was then coated and heated to prepare high-performance PI membranes for separating helium from natural gas or other systems. However, the trade-off effect between permeability and selectivity remains a challenge for PI membranes. In addition, polyimides with relatively low glass transition temperatures (*T*_g_) exhibit a higher polymer chain mobility at lower operating temperatures, which makes these polyimides susceptible to plasticization and, thus, undermines membrane selectivity [13]. To address these issues, global researchers have focused on enhancing the gas separation performance and anti-plasticization property of PI membranes through modification strategies like thermal rearrangement, chemical cross-linking, and physical blending [14,15,16].

**Figure 1:**
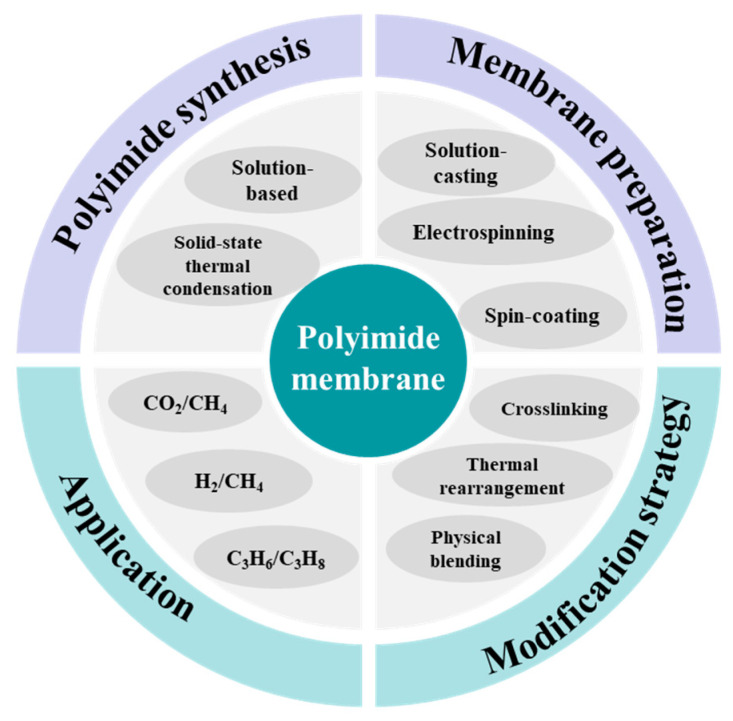
Synthesis, preparation, and modification of polyimide membranes for gas separation.

In this review, we critically summarize the synthesis methods of PI materials, preparation and modification strategies for PI membranes, and their applications in gas separation (Figure 1). Firstly, various diamine and dianhydride monomers employed in synthesizing PI for gas separation are described. Subsequently, the different polymerization routes (solution-based method and solid-state thermal condensation method) for PI synthesis and the common fabrication methods (solution-casting, spin-coating, and electrospinning) of PI membranes are elaborated. Additionally, modification strategies for polyimide membranes are discussed, including thermal rearrangement, cross-linking modification, and physical blending for the preparation of mixed matrix membranes (MMMs). The main advances of polyimide membranes in gas separation, such as CO_2_/CH_4_ separation, H_2_/CH_4_ separation, and propylene/propane separation, are thoroughly discussed. Finally, this review outlines the existing challenges, anticipated prospects, and further research expectations in this area.

## 2. Synthesis of Polyimide

The pioneering synthesis of aromatic polyimides dates back to Bogert and Renshaw in 1908, who achieved the intramolecular polycondensation of 4-amino-o-phthalic acid [17]. Over a century of subsequent advancement, extensive combinations of dianhydrides and diamine monomers, along with diverse synthetic methodologies, have not only led to the creation of polyimides in various structural forms, but also established the synthesis of polyimides as a broad research area. Based on their main-chain structural features, polyimides can be classified into the following three principal categories: fully aromatic, alicyclic, and fluorine-containing types [18,19,20]. From a synthetic perspective, the principal preparation methods for PIs are categorized into solution-based methods and solid-state thermal condensation methods.

### 2.1. Monomer Structures

**Figure 2:**
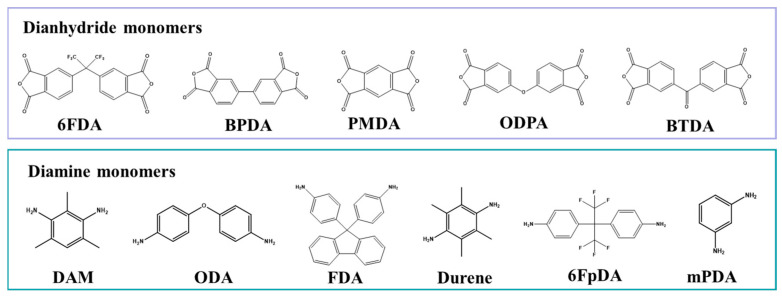
Dianhydride/diamine monomer structures employed in typical PI synthesis.

A significant number of monomers can be used in PI synthesis. The physicochemical properties of PI can be extensively modified through minor alterations to its backbone structure. Monomers are classified into the following three categories based on their chemical structure: non-coplanar (kink, spiro, and cardo) monomers, symmetric/asymmetric monomers, and fluorinated monomers [10]. The selection of monomers is guided by criteria such as rigidity, symmetry, bulkiness, and the introduction of functional groups. The use of rigid and symmetrical aromatic monomers enhances molecular chain regularity and π-π stacking interactions, which significantly elevates the glass transition temperature [21]. Conversely, introducing aliphatic or flexible linking groups reduces chain rigidity, thereby lowering heat resistance [22]. Consequently, the selection of appropriate diamine and dianhydride monomers, whose chemical structures ultimately dictate polyimide properties, including thermal stability, mechanical strength, and gas transport characteristics, is critical for tailoring polyimides with a targeted performance. Figure 2 illustrates the multiple dianhydride and diamine monomers employed in PI synthesis. Aromatic dianhydrides, such as pyromellitic dianhydride (PMDA), 4,4′-(hexafluoroisopropylidene)-diphthalic anhydride (6FDA), 3,3′,4,4′-oxydiphthalic anhydride (ODPA), 3,3′,4,4′-biphenyltetracarboxylic dianhydride (BPDA), and 3,3′,4,4′-benzophenonetetracarboxylic dianhydride (BTDA), are commonly employed to construct the rigid backbone of polyimides, endowing the materials with a favorable thermal stability and mechanical strength [23]. Saeed et al. [24] investigated the characteristics of polyimide derived from dianhydrides containing distinct bridging groups, ODPA and BPDA. It was discovered that the molecular weight distribution of the resulting polyimide was influenced by the electron affinity of the dianhydride component. More specifically, the ODPA-based polyimide exhibited a higher content of low-molecular-weight species when compared to that synthesized from BPDA. Chen et al. [25] synthesized polyimides using 6FDA as a monomer, achieving an excellent gas separation performance. The incorporation of flexible substituent groups in the polymer backbone enhances processability, while the presence of bulky and polar groups (CF_3_) improves gas permeability [26].

In addition, the impacts of diamine monomers on the characteristics of PI have also been explored. For instance, 2,4,6-trimethyl-m-phenylenediamine (DAM), 4,4′-oxydianiline (ODA), 9,9-bis (4-aminophenyl) fluorene (FDA), and 2,3,5,6-tetramethyl-1,4-phenylenediamine (Durene) have attracted much attention due to their rigid and accessible structures [27,28]. FDA, with a cyclic cardo group, has a high solubility in solvents, which makes it suitable for the preparation of gas separation membranes. The incorporation of asymmetrical DAM within the polymer backbone can enhance the diffusion of permeating molecules through the polymer matrix. Dingemans et al. [29] found that changes in the chemical structure of diamine monomers (such as the substitution position of the aryl ether linking group) significantly alter the crystallization ability of polyimides. Para-substituted diamines form semi-crystalline polymers, while meta-substituted diamines form amorphous polymers. In another report, Seo et al. [30] found that the crystallinity of BPDA-ODA polyimide is much lower than that of BPDA-PDA polyimide. This is because the flexible ether linkage in ODA disrupts chain linearity and reduces the rigidity of the polymer backbone, thereby inhibiting efficient chain packing and crystallization. Utilizing various diamines, Qiu et al. [31] prepared a series of 6FDA-based polyimides. Selecting specific diamine monomers enables effective modulation of the chain packing density and free volume in the resulting PI membranes. In summary, the high-throughput screening of dianhydride and diamine monomers, coupled with precise structural modulation, provides an effective methodology for engineering polyimides to meet diverse application requirements. However, the chemical structures of monomers are highly diverse, and their combinations can form a vast number of candidate systems. To avoid the time-consuming and inefficient process of traditional trial-and-error experiments to screen for the optimal monomer, Wang et al. [32] screened high-performance polyimides from a large number of candidates through machine learning. Although monomer design provides space for performance tuning, the inherent properties of monomers also bring challenges to practical applications, such as the poor solubility of rigid aromatic dianhydrides along with their high melting point. In order to overcome the synthetic obstacles posed by monomers and to realize the synthesis of high-quality polyimides, it is crucial to employ appropriate synthetic methods.

### 2.2. Polyimide Synthesis

#### 2.2.1. Solution-Based Method

Solution-based PI synthesis primarily employs one-step and two-step approaches. The former directly synthesizes PI from dianhydride and diamine monomers in high-boiling solvents at elevated temperatures. In this process, polycondensation and dehydration cyclization proceed concurrently, and the formation of polyamic acid is bypassed. Severe reaction conditions significantly impede solvent recovery and elevate energy consumption. Compared with the one-step approach, the two-step approach is more commonly employed for polyimide synthesis, as established in previous studies [25,33,34,35,36]. The conventional two-step polycondensation technique is exemplified by the industrial production of DuPont’s Kapton^®^ membranes. In this process, amine and anhydride monomers first undergo ring-opening polyaddition reactions in aprotic solvents (such as N, N-dimethylformamide (DMF) and N-methyl-2-pyrrolidone (NMP)), resulting in the formation of polyamide acid (PAA) prepolymers. Subsequently, the resulting PAA precursors are subjected to further condensation through imidization reactions; meanwhile, the by-product water is eliminated through azeotropic distillation or supplementary thermal processing to enhance the condensation levels. Common dehydrating agents include anhydrides and other organic acid derivatives, and catalysts contain triethylamine and pyridine, etc. A suitable catalytic dehydration system should be selected based on the reaction conditions to avoid the formation of thermosensitive isocyanimides. This method offers notable benefits, for example, it is highly adaptable to diverse monomers, especially for constructing aromatic polyimides. Additionally, the mild reaction temperature prevents the thermal degradation of monomers and polymers [37].

Building upon the established solution-based methods, significant research focus has recently shifted towards exploring solvent/hydrothermal methods for polyimide synthesis [38,39]. This approach enables controllable polymerization under ultra-boiling point conditions through a closed reaction system, such as an autoclave. The effectiveness of this method stems from the unique high-pressure, high-temperature aqueous environment employed. High-pressure hydrothermal conditions facilitate the stepwise progression of dissolution, polymerization, and crystallization, thereby enhancing the reaction kinetics. Notably, the Unterlass group [40] developed an eco-friendly hydrothermal approach employing water as the reaction medium. This method does not require organic solvents or additional catalysts, aligning with green chemistry principles and offering a novel strategy for synthesizing environmentally friendly polyimides.

#### 2.2.2. Solid-State Thermal Condensation Method

**Figure 3:**
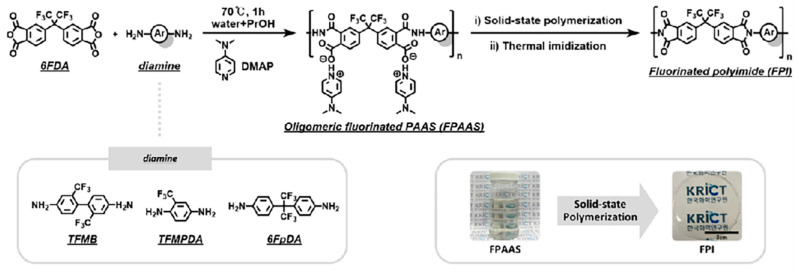
Synthetic schematic diagram of 6FDA-based FPIs. Reproduced with permission from ref. [41]. Copyright 2024, Elsevier B.V.

Solid-state thermal condensation enables polyimide synthesis through direct heat-induced polymerization by physically blending amine and anhydride monomers, eliminating solvent usage. Upon heating to a temperature near the melting point of the anhydride monomer, the anhydride melts and reacts with the neighboring amine monomer to form oligomers. Subsequently, driven by intermolecular interactions, these oligomers undergo further polymerization to generate long polymer chains. Park et al. [41] resolved the issue of fluorinated monomer solubility through the water/alcohol co-solvent system, which facilitated the solid-state polymerization and thermal imidization process via stepwise heating (Figure 3). This approach is environmentally friendly, cost-effective, and scalable, as it eliminates the need for toxic solvents. However, it requires high temperatures and a prolonged reaction time, driving the development of more efficient methods.

According to previous studies, Zhou et al. [42] reported a rapid synthesis strategy using microwave-assisted heating for preparing polyimide. Through microwave-assisted thermal treatment, polyimide achieved a substantially higher imidization degree at 250 °C. This value was approximately double that attained using conventional thermal imidization protocols. Moreover, when processed at 300 °C via microwave-induced imidization, the resulting PI membranes exhibited tensile strength and elastic modulus enhancements of roughly 30% relative to counterparts prepared by standard thermal methods. These improvements are likely attributed to a more uniform distribution of the heat field, which reduced the thermal stress gradient. These findings collectively demonstrate that microwave-assisted imidization represents a rapid and highly efficient pathway for fabricating high-performance polyimide materials.

In addition to the above methods, vapor deposition is also used in polyimide synthesis. Ultra-thin polyimide coatings can be deposited on the substrate surface through chemical vapor deposition, enabling precise control of the nanoscale thickness [43]. Among them, the solution-based condensation method serves as one of the most mature PI synthesis routes in gas separation. Furthermore, in comparison with the solid-state thermal condensation method, solution-based polycondensation occurs at a lower reaction temperature. The removal of small-molecule by-products can be effectively accomplished through the use of an azeotropic solvent. The incorporation of such a solvent not only enhances the absorption of reaction heat, but also ensures the efficient and stable progression of the reaction.

## 3. Fabrication and Modification of Polyimide Membranes

### 3.1. Fabrication Strategies

**Figure 4:**
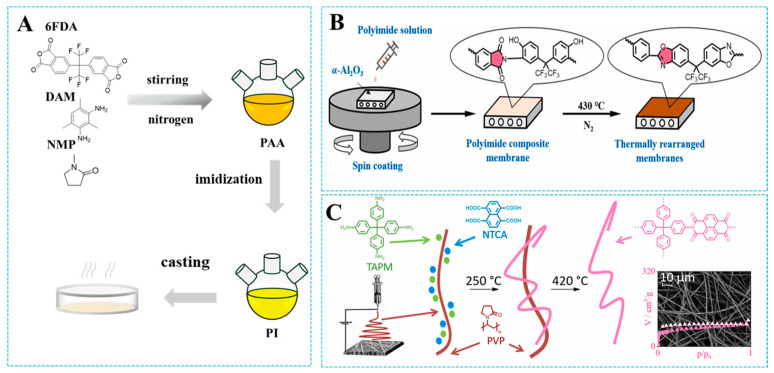
Different membrane-fabricating methods: (**A**) Solution-casting. (**B**) Spin-coating. Reproduced with permission from ref. [51]. Copyright 2024, Elsevier Ltd. (**C**) Electrospinning. Reproduced with permission from ref. [53]. Copyright 2021, The Authors. Published by Elsevier Inc.

Polyimide membranes are mainly fabricated through techniques like solution-casting, spin-coating, and electrospinning (Figure 4), with each method providing unique benefits and facing specific challenges [44,45,46]. The solution-casting method entails polymer dissolution in a volatile solvent to form a homogeneous solution, with subsequent degassing and casting onto a flat substrate. A membrane is finally prepared by evaporating the solvent. This approach features operational simplicity and favorable cost characteristics, rendering it suitable for large-scale production. Moreover, solution-casting prevails as the predominant fabrication technique in contemporary studies of polyimide gas separation membranes [47]. For example, Xie et al. [48] dissolved 6FDA-DAM into NMP to form a uniform solution, and then the solution was poured into a clean glass substrate at 80 °C to evaporate the solvent. However, traditional solvents (such as NMP and DMF) have environmental toxicity problems. In response to this problem, researchers have recently proposed a strategy of using green solvents. Rasool et al. [49] selected gamma-Valerolactone (GVL) as a renewable solvent for the preparation of PI membranes. The low toxicity and biodegradability of GVL significantly reduced ecological impacts.

While solution-casting enables the cost-effective scale-up of PI membranes, its limited thickness uniformity restricts applications requiring nanoscale precision. In contrast, the spin-coating method can prepare a thin and uniform membrane. Spin-coating employs solution deposition onto substrates followed by rapid rotation to achieve uniform liquid spreading [50]. Subsequently, the resulting liquid layer solidifies to create a homogeneous and thin membrane after drying. For instance, Zhou et al. [51] fabricated polyimide-based membranes on α-Al_2_O_3_ substrates using spin-coating, investigating selective layer thickness and integrity through modulation of the coating solution concentration. This technology has become a commonly used method in laboratories due to its high uniformity and simple process, but its high material waste and limited substrate adaptability have restricted its industrial application.

Electrospinning technology shows great potential for gas separation, as it can produce nanofiber membranes featuring elevated specific surface areas and tailorable pore architectures. Unlike dense membranes, nanofiber membranes function primarily as depth filters, where gas transport occurs through voids between fibers rather than solution diffusion through the polymer matrix. Therefore, their selectivity is lower than that of dense membranes [52]. Electrospinning utilizes a high-voltage electrostatic field to generate polymer jets, which undergo field-driven stretching and solvent/melt phase transitions to form nanofiber membranes with tunable porous morphologies [52]. Breunig et al. [53] adopted electrospinning technology and transformed monomeric precursors into self-standing membranes composed of porous organic polymers, with a CO_2_ adsorption capacity reaching 3.0 mmol/g and CO_2_/CH_4_ selectivity of 20. This further indicates that the application of porous polymers in gas separation is more accessible. However, this technique suffers from a compromised mechanical strength due to weak fiber–fiber interactions. Given the above limitations, current research efforts remain predominantly focused on tailored process innovation for targeted membrane applications to enhance scalability and structural stability through systematic process optimization. The performance of polyimide membranes is not only determined by their chemical structure, but is also significantly influenced by the thermal and mechanical history experienced during the manufacturing process. Key process parameters are pivotal in shaping the membrane’s microstructure. The thermal imidization protocol (heating rate and curing temperature) and mechanical stretching procedures directly influence the evolution of critical features such as the degree of imidization, polymer chain orientation, and crystallization. Huo et al. [54] studied the effect of uniaxial stretching on the gas separation performance of PI. As stretching progressed, due to dense chain-like stacking, PI membranes showed a decrease in gas permeability and an increase in gas selectivity after stretching treatment. Therefore, systematic control of these preparation parameters can further enhance the performance of polyimide membranes.

### 3.2. Modification Strategies

Despite their advantages of high selectivity and excellent thermal and chemical stability, polyimide membranes still suffer from low permeability and insufficient resistance to plasticization. These limitations escalate investment costs and undermine economic viability. Especially when employed for CO_2_ separation, the fractional free volume (FFV) structure of PI membranes is destroyed under high-concentration CO_2_ exposure, which leads to plasticization [55]. This phenomenon enhances the segmental mobility of polymer chains while compromising molecular sieving capacity, ultimately elevating gas permeability but diminishing selectivity [56]. Consequently, it is essential to modify PI membranes to enhance their physicochemical stability and achieve a superior gas permeability, ultimately leading to greater economic, social, and ecological benefits [57]. To this end, researchers have optimized membrane performance through diverse strategies, including thermal rearrangement, cross-linking, and blending modification.

#### 3.2.1. Thermal Rearrangement

Thermally rearranged (TR) polymers have emerged as promising candidates for gas separation, particularly in CO_2_/CH_4_ systems, due to their exceptional separation performance and inherent resistance to plasticization [58,59]. The strategy of thermal rearrangement is based on the solution processability of soluble precursors and the controllable thermally induced phase transition mechanism. Specifically, after the precursor solution is processed into a membrane, the topological rearrangement and cyclization of the polymer chains are triggered through temperature programming treatment. TR polymers display an outstanding gas separation performance attributable to the formation of rigid polybenzoxazole (PBO) architectures during thermal conversion. This performance is further enhanced by the formation of a bimodal cavity size distribution: cavities around 0.3–0.4 nm provide molecular sieving characteristics, while larger cavities around 0.7–0.9 nm enable fast gas permeation [60]. In addition to their outstanding gas transport, TR polymers also demonstrate an excellent resistance to plasticization against high-pressure condensed gases (such as CO_2_) due to their inherent rigid structure [61,62].

**Figure 5:**
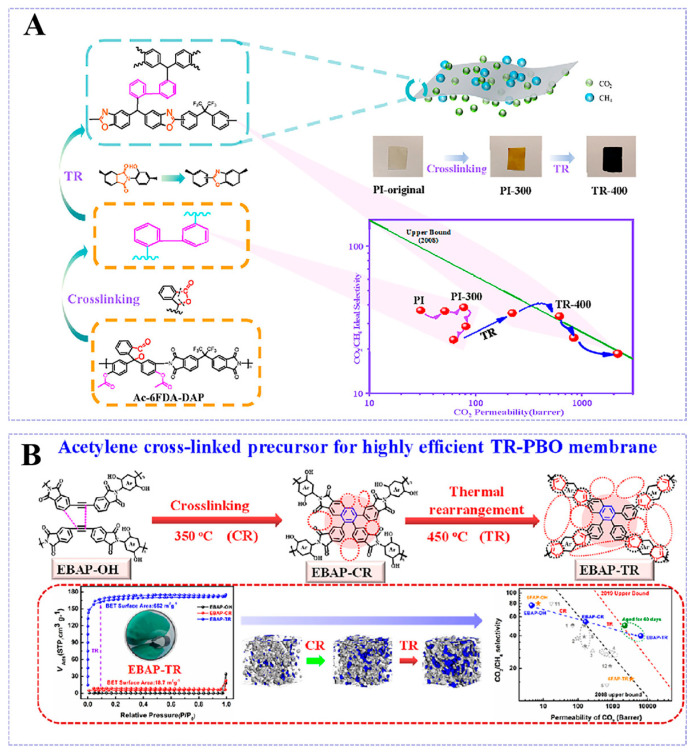
(**A**) Schematic illustration of the thermal cross-linking and thermal rearrangement reactions in AC-6FDA-DAP polyimide. Reproduced with permission from ref. [64]. Copyright 2022, Elsevier B.V. (**B**) Fabrication and architectures of: o-hydroxy-functionalized acetylene-polyimide (EBAP-OH), self-crosslinking polyimide networks (EBAP-CR), and thermally rearranged polybenzoxazole (EBAP-TR). Reproduced with permission from ref. [65]. Copyright 2023, Elsevier B.V.

Over recent decades, thermally rearranged membranes have attracted significant research attention for gas separation. Park et al. [63] pioneered TR polymers synthesized via the thermal rearrangement of ortho-functionalized hydroxyl-containing polyimides (α-OH-PIs) for CO_2_/CH_4_ separation, which exhibited a high resistance to plasticization induced by CO_2_. The chemical architecture of precursors also has an effect on the gas separation performance of TR membranes. Du et al. [64] developed polyimide precursors with the incorporation of cardo-structured phenolphthalein. A PBO polymer was formed through thermal arrangement. This thermal rearrangement occurred under 400 °C, where the imine ring was converted into a benzoxazole microporous structure, further expanding the d-spacing to 6.16 Å. This process resulted in a significant increase in free volume, effectively improving CO_2_ solubility in the membrane, which enhanced the CO_2_ permeability of the TR-400 PBO membrane (Figure 5A). With the heating temperature increased from 275 °C to 400 °C, the CO_2_ permeability of the TR membranes increased significantly from 50.9 Barrer to 613 Barrer. In addition, the TR membranes demonstrated a good plasticization resistance under 30 atm pressure for 24 h. Zhang et al. [65] designed EBAP-TR membranes by the thermal rearrangement of polymers at 450 °C (Figure 5B). This process prompted the conversion of the o-hydroxyimine ring into a benzoxazole rigid ring at 450 °C, increasing the BET surface area of the material to 662 m^2^/g, along with a free volume fraction of 0.27. The EBAP-TR membranes displayed a super-high CO_2_ permeability of 6455 Barrer and CO_2_/CH_4_ selectivity of 40. Furthermore, the activation energy difference of 7.42 kcal/mol for the EBAP-TR membranes between CO_2_ and CH_4_ also indicated a high CO_2_/CH_4_ separation performance. Hu et al. [66] developed copolymer membranes by blending TR-able o-hydroxyl polyimides with non-TR-able polyimides incorporating rigid Tröger’s Base (TB) units. The synergistic interactions between the TR-converted domains and the TB structural segments improved the rigidity and microporosity of the polyimides, which increased gas sorption within the free volume in the polymer matrix. Consequently, the developed TR polyimide membranes achieved excellent gas transport properties, exceeding the 2008 Robeson upper bound.

**Figure 6:**
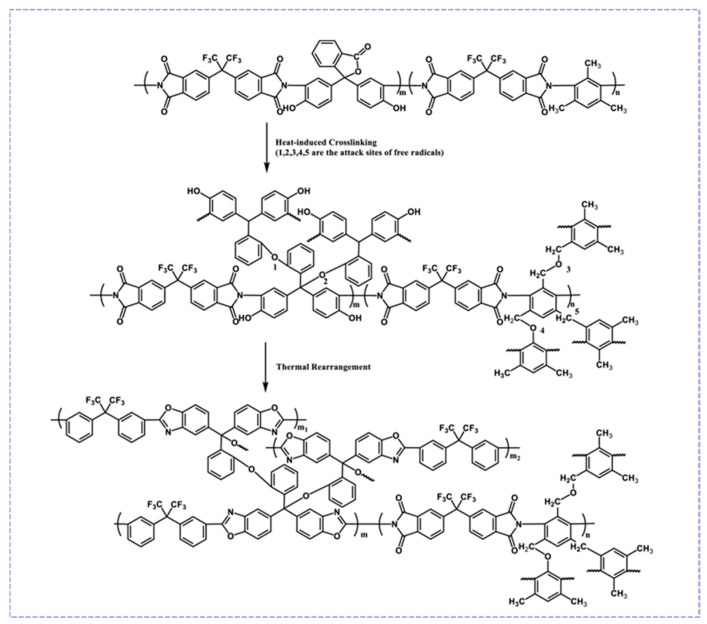
Crosslinking and thermal rearrangement reactions of Co-PI-DAM. Reproduced with permission from ref. [67]. Copyright 2024, American Chemical Society.

However, elevated thermal rearrangement temperatures frequently induce structural collapse in the microporous architecture of asymmetric polymeric membranes, consequently increasing the gas mass transfer resistance. As shown in Figure 6, Wang et al. [67] prepared cross-linked TR polymer membranes from copolymer polyimides (Co-PI-DAM/DETDA). The introduction of bulky diamines (DAM/DETDA) via copolymerization aimed to form a hydrogen-bonded network with DAP. This approach significantly increased the high glass transition temperatures of the precursor, so thermal cross-linking (300–350 °C) and TR (400–425 °C) were both achieved at sub-*T*_g_. The cross-linked network inhibited the movement of chain segments at high temperatures to avoid pore collapse. In conclusion, the TR process significantly improved the gas permeability and anti-plasticization property of membranes. These findings indicate that future research shall focus on both developing strategies to lower TR operational temperatures and systematically investigating the structure–property relationships between membranes and their separation performance.

#### 3.2.2. Cross-Linking Modification

Cross-linking modification constitutes a prevalent strategy in PI membrane modification for effectively mitigating the plasticization process in gas separation. This approach facilitates cross-linking reactions within polymer chains, thereby decreasing chain motion and significantly restricting membrane swelling induced by condensable gases. After cross-linking modification, polyimide chains can form a three-dimensional network structure. By restricting the movement of polyimide chains, the separation performance of the membrane is improved, which causes a decrease in gas permeability and an increase in selectivity [15]. Certain cross-linked networks arise from physical bonds (e.g., hydrogen bonds), whereas others derive from chemical bonds [68,69]. Diverse methods facilitate the chemical bonding of polymer chains, including ultraviolet (UV) cross-linking, chemical cross-linking, and thermal cross-linking [70].

In 1994, Kita et al. [71] reported an ultraviolet-induced cross-linking phenomenon in PI membranes prepared via the polycondensation of BTDA or BTDA/6FDA with TMPD. After 30 min of ultraviolet irradiation, the selectivity of H_2_/CH_4_ increased by 50 times, while the permeability of H_2_ decreased. In addition, as the duration of ultraviolet irradiation extended, the permeability coefficient of H_2_ in the membrane decreased, whereas the permeation selectivity increased. Wu et al. [72] reported a novel approach via introducing isophthalic dihydrazide as a physical cross-linking agent into polyimides to design hydrogen bonds on the polymer chains, which can enhance the interactions between polymer chains to regulate chain packing. Since the hydrazide group was much less basic than the amino group and was unable to attack the imide group, no chemical reaction occurred in the system, thus preserving the intact chemical structure of the Du-PI membrane. The cross-linked membranes demonstrated an enhanced mechanical stability and improved plasticization resistance, while achieving a 505% enhancement in H_2_/CH_4_ selectivity when compared to pristine polyimide membranes.

**Figure 7:**
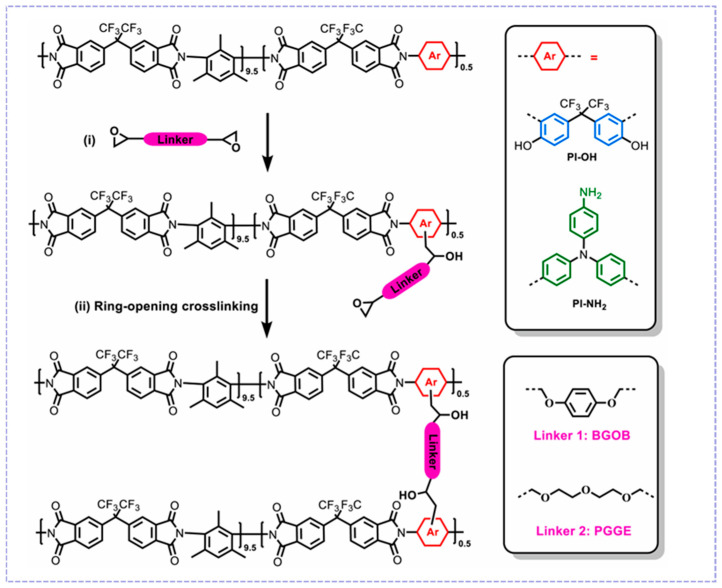
Crosslinking of PI-OH/PI-NH_2_ with diepoxide crosslinkers. Reproduced with permission from ref. [76]. Copyright 2024, Elsevier B.V.

In addition to the physical cross-linking mentioned above, chemical cross-linking has also been used to enhance membrane separation performance [73]. Diol cross-linking, a common chemical cross-linking method, forms an ester bond by reacting the hydroxyl groups of diols with the side-chain carboxyl groups in PI [74]. For example, Esteban et al. [75] used 1,4-butanediol as a cross-linking agent to modify a copolymer containing carboxyl groups with a high polarity. Their work employed copolymerization with carboxyl-containing DABA diamine, enabling chemical cross-linking of the polymer material via carboxyl sites. The chemical cross-linking enabled membranes not only to exhibit good mechanical properties, but also to enhance their anti-plasticization ability. Yang et al. [76] studied the effect of two diepoxide cross-linkers on the structure of polyimides, respectively (Figure 7). A BGOB cross-linker containing a bulky benzene ring structure created significant spatial site resistance during cross-linking, preventing the polymer chains from tightly stacking (d-spacing increased from 6.33 Å to 6.55 Å), thus resulting in a significant increase in the O_2_/N_2_ selectivity of the PI-OH-BGOB membrane. The strong quadrupole-dipole interaction of another cross-linker, PGGE, via its ether bond with CO_2_, enhanced CO_2_ solubility selectivity. Thus, the CO_2_/N_2_ selectivity of the PI-OH-PGGE membrane increased to 32.7. In addition, the cross-linked membranes exhibited exceptional anti-aging and excellent stability under high-pressure conditions ranging from 2 bar to 12 bar. The cross-linked covalent network effectively resisted high-pressure CO_2_-induced swelling.

**Figure 8:**
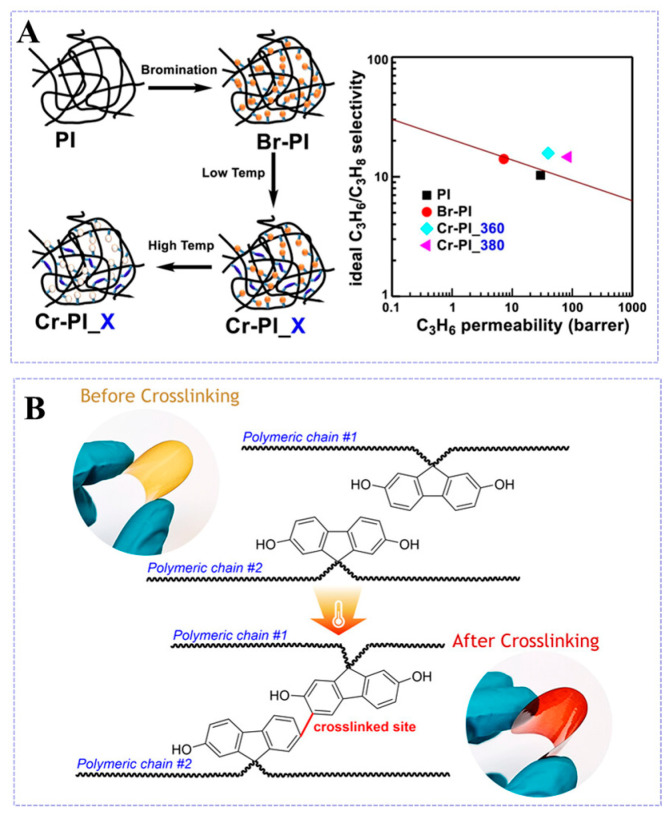
(**A**) Schematic illustration of thermal cross-linking of Brominated 6FDA-DAM. Reproduced with permission from ref. [78]. Copyright 2021, Elsevier B.V. (**B**) Schematic representation of thermal cross-linking structure of the 6FDA-Durene/CARDO(OH) (3:1) block copolyimide. Reproduced with permission from ref. [79]. Copyright 2024, American Chemical Society.

In certain cases, thermal annealing also results in the cross-linking of PI membranes [77]. Do et al. [78] designed polyimide membranes by heat-induced chemical cross-linking (Figure 8A). Bromine atoms were first introduced into the main chain of 6FDA-DAM to form brominated polyimide, and then thermally induced debromination cross-linking was carried out at different temperatures ranging from 250 °C to 380 °C to obtain thermally cross-linked membranes. Nitrogen adsorption/desorption isotherms revealed pronounced microporosity in the Cr-PI_380 membrane, evidenced by a BET surface area of 534 m^2^/g. This elevated surface area correlated with an enhanced free volume, directly improving gas permeability. Notably, as prepared by thermal treatment at 380 °C for 5 h, the Cr-PI_380 membrane demonstrated the optimal propylene permeability (84.70 Barrer) and propylene/propane selectivity (14.6), while also showing an excellent resistance to plasticization. Heating duration also has an effect on the gas separation performance of PI membranes. Hayek et al. [79] developed 6FDA-Durene/CARDO(OH) (3:1) copolyimide membranes and investigated the effect of heating duration on the CO_2_/CH_4_ separation performance of these copolyimide membranes. A diagrammatic illustration of the thermal cross-linking structure for the copolyimides is shown in Figure 8B. The color of the resulting cross-linked membranes changed from yellow to reddish brown and was insoluble in organic solvents, demonstrating successful cross-linking. The thermal cross-linking process caused the polymer chains to come closer together, resulting in a reduction in FFV, decreasing CO_2_ permeability. Meanwhile, the restricted movement of the chain segments resulted in an 18.7% increase in CO_2_/CH_4_ selectivity compared to uncross-linked membranes. The findings revealed that as the thermal cross-linking time increased, the CO_2_ permeability coefficient decreased while the CO_2_/CH_4_ selectivity improved.

Different cross-linking modification approaches have demonstrated their respective advantages in improving the gas separation performance and anti-plasticization property of polyimide membranes. These studies have provided a solid theoretical foundation and systematic practical guidance for the development of high-performance polyimide membranes. Future research can further explore the synergistic effects among different chemical cross-linking methods, thereby achieving further improvement in the separation performance of polyimide membranes.

#### 3.2.3. Physical Blending

Similar to most polymer membranes, polyimide membranes have an inherent trade-off effect between permeability and selectivity, which restricts their further development in industrial gas separations. The physical blending of a polymer matrix and inorganic fillers to prepare MMMs is regarded as a promising method to address this trade-off effect [80]. Mixed matrix membranes integrate the superior mechanical properties of polymeric membranes with the gas sieving and rapid mass transfer capabilities of nanofillers. MMMs can achieve both high permeability and selectivity, particularly when the fillers exhibit a strong affinity towards a specific gas. This strategy has been employed to improve CO_2_ permeability through the addition of an amine-functionalized metal–organic framework (MOF) possessing a high CO_2_ affinity [81].

**Figure 9:**
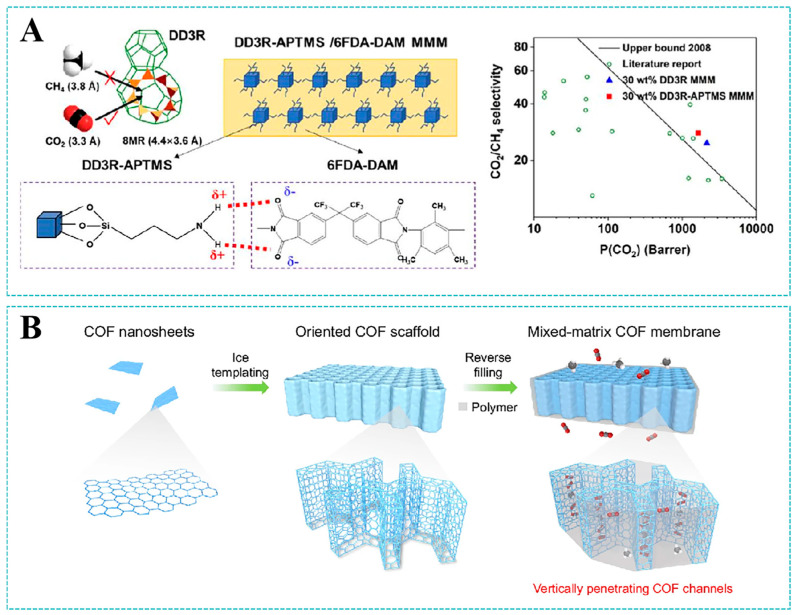
(**A**) Schematic illustration of 6FDA-DAM MMMs incorporating surface-modified DD3R nanoparticles for CO_2_/CH_4_ separation. Reproduced with permission from ref. [83]. Copyright 2024, Elsevier B.V. (**B**) Schematic illustration of the reverse-filling approach to fabricating MMMs with continuous and vertically penetrating COF channels for CO_2_/CH_4_ separation. Reproduced with permission from ref. [86]. Copyright 2025, The Authors.

Datta et al. [82] successfully engineered MMMs by embedding AlFFIVE-1-Ni MOF nanosheets into a polyimide matrix. Regulating the morphology of MOF crystals along the (001) crystal direction provided the maximum one-dimensional molecular channel exposure for the formation of high-aspect-ratio (001) nanosheets. This promoted interactions between nanosheets and polymers, achieving high filler loadings of 60 wt%. Gas diffused preferentially along one-dimensional channels, which demonstrated a better selectivity for CO_2_/CH_4_. Specifically, when the loading of fillers was 60.3%, the AlFFIVE-1-Ni MOF/6FDA-DAT MMMs exhibited a CO_2_/CH_4_ selectivity of 122.1. Chen et al. [83] simultaneously enhanced CO_2_/CH_4_ separation performance and plasticization resistance in 6FDA-DAM membranes via surface-modified DD3R zeolite nanoparticles. Figure 9A illustrates that the nanofillers created CO_2_-selective channels while restricting polymer chain mobility through hydrogen-bonding networks between APTMS amino groups and 6FDA-DAM carboxyl moieties. Incorporating 30 wt% DD3R-APTMS elevated CO_2_ permeability by 71.7% and CO_2_/CH_4_ selectivity by 44.5% versus pristine membranes.

However, the weak physicochemical properties between polymer and inorganic filler leads to a poor interfacial compatibility, which remains a critical challenge for mixed matrix membranes. So far, many studies have proposed favorable approaches for MOF-based MMMs to enhance the compatibility between MOFs and polymers, thereby minimizing interfacial defects. For example, Essen et al. [84] prepared MMMs by incorporating ZIFs and two polymers (Matrimid^®^ 5218 and polybenzimidazole oPBI, Matrimid^®^ 5218 was supplied by Huntsman (Basel, Switzerland) and PBI was obtained from Fumatech (Bietigheim-Bissingen, Germany)). The excellent compatibility between the polymer matrix and the ZIFs suppressed the formation of non-selective defects, thereby increasing the CO_2_/N_2_ separation factor. In another study, Zhao et al. [85] developed carboxyl-functionalized copolyimide (6FBDA) to improve interfacial compatibility with aminated ZIF-8. The structural similarity between imidazole units of 6FBDA and ZIF-8-NH_2_ ligands facilitated π-π stacking, while carboxyl groups formed hydrogen bonds with amino ligands of ZIF-8-NH_2_, both greatly enhancing the interfacial compatibility between the polymer and MOF. When the loading of nanofillers reached 50 wt%, the MMMs exhibited invisible interface defects. This provides a new method for reducing interface defects between the polymer matrix and nanofillers.

Jia et al. [35] integrated two-dimensional ZIF-L nanosheets into a polyimide matrix to prepare MMMs. The ordered arrangement of ZIF-L in the polymer matrix provided a rapid transport channel for H_2_, which caused an improvement in H_2_/CO_2_ selectivity. They also systematically investigated the influence of three distinct coating methods (casting, flat scraping, and spin coating) on the separation performance of membranes. It was revealed that spin-coated MMMs with 45 wt% filler demonstrated a superior performance, achieving a H_2_ permeability of 368.9 Barrer. Generally, MMMs are fabricated through the processes of solution casting and solvent evaporation. Different from previous membrane-fabricating methods, Guo et al. [86] employed an innovative reverse filling technique to prepare MMMs (Figure 9B). Covalent organic framework (COF) nanosheets with a vertical orientation were constructed through the ice templating method, then filled with polymers in the reverse direction. The continuous and vertically penetrating COF channels formed in the membrane significantly shortened the gas transport pathways and preserved the inherent CO_2_-philic pore structure of the COF. The unique structural features of the COF scaffold enabled membranes with a high CO_2_ permeability of 972 Barrer, CO_2_/CH_4_ selectivity of 58, and excellent long-term stability.

**Figure 10:**
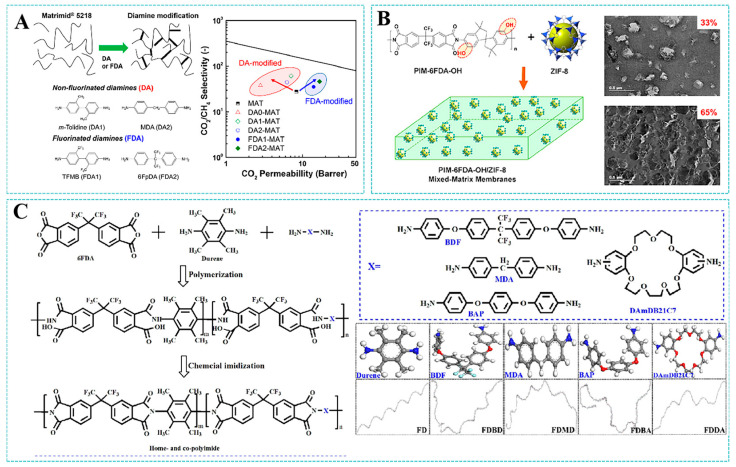
(**A**) Diamine modification of polyimide membranes. Reproduced with permission from ref. [87]. Copyright 2022, The Authors. (**B**) Hydroxyl-functionalized PIM. Reproduced with permission from ref. [88]. Copyright 2018, American Chemical Society. (**C**) The synthesis of co-polyimides. Reproduced with permission from ref. [89]. Copyright 2023, Elsevier B.V.

In addition to the modification strategies mentioned above, there are also numerous research reports on improving the gas separation performance of PI membranes via strategies such as the molecular design of monomers, grafting additional groups onto existing polymers, the copolymerization of polymers, etc. As shown in Figure 10A, Lee et al. [87] examined the effects of diamine structure on the separation performance and physicochemical properties of polyimide membranes. Modification with bulky fluorinated diamines introduced fluorinated groups that optimized polymer chain packing. This structural rearrangement generated enlarged free volume elements, consequently enhancing gas permeation. As a result, compared to the commercial PI membrane, the modified Matrimid^®^ 5218 membranes exhibited synergistic enhancements in H_2_ permeability and H_2_/CH_4_ separation selectivity. Ma et al. [88] developed MMMs by blending hydroxyl-functionalized microporous polyimide PIM-6FDA-OH with ZIF-8. The strong N···O-H hydrogen bonding interaction between the polymer’s hydroxyl groups and the imidazole nitrogen of the MOF enhanced interfacial compatibility, achieving a high filler loading up to 65 wt% without invisible interfacial defects. The MMMs exhibited a separation factor of 43 for propylene/propane, and the permeation flux of propylene was ten times greater than that of the pure membrane when the loading of ZIF-8 filler reached as high as 65% (Figure 10B). Wu et al. [89] synthesized different copolyimide membranes by integrating four different diamine monomers into 6FDA-Durene-based polyimide (Figure 10C). The specific geometry and functional groups of these diamine monomers (particularly the crown ether structure) significantly reduced the average d-spacing, narrowed the cavity size distribution, and minimized large free volume elements. This engineered microstructure, characterized by an enhanced molecular sieving capability, dramatically improved gas diffusion selectivity. The CO_2_/CH_4_ diffusion selectivity increased by up to 136.8% for the 6FDA-Durene-based polyimide membrane, introducing a crown ether structure.

## 4. Applications in Gas Separation

Polyimide membranes play a crucial role in molecular separation owing to their outstanding mechanical properties, high thermal stability, and excellent chemical durability under harsh conditions. More importantly, polyimide membranes can be employed in numerous industrial gas separation applications, including the removal of CO_2_ from CH_4_, the recovery of H_2_ from CH_4_, and the separation of C_3_H_6_/C_3_H_8_. According to studies reported in recent years, aromatic polyimides containing 6FDA are characterized by the presence of CF_3_ bulky groups, which contribute to an increased free volume within the polymer, enabling effective gas transport through polymer matrices [90,91,92]. Thus, the 6FDA-based polyimide membrane exhibits a superior gas permeability and has recently been extensively researched in gas separation applications.

### 4.1. CO_2_/CH_4_ Separation

As an eco-friendly, dependable, and cost-effective energy resource, natural gas plays a vital role in addressing the steadily rising global demand for energy. The CO_2_ present in raw natural gas induces pipeline corrosion and reduces calorific value, making efficient CO_2_/CH_4_ separation critical for purification processes to ensure pipeline integrity and maintain gas quality [93].

**Figure 11:**
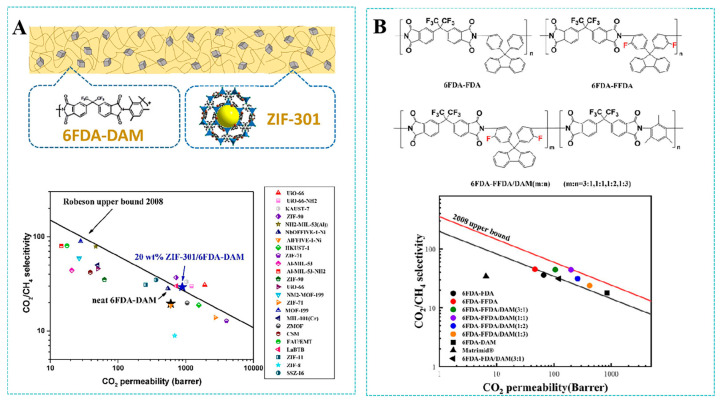
(**A**) ZIF-301/6FDA-DAM MMMs for CO_2_/CH_4_ separation. Reproduced with permission from ref. [96]. Copyright 2021, Elsevier B.V. (**B**) CO_2_/CH_4_ separation performance of 6FDA-FFDA/DAM membranes. Reproduced with permission from ref. [98]. Copyright 2023, Elsevier B.V.

In natural gas purification processes, PI membranes are not only limited by CO_2_-induced plasticization, but also by the trade-off effect between permeability and selectivity, which limits industrial application [94]. Existing studies have demonstrated that crosslinked polymers and the incorporation of functional nanofillers represent viable strategies to effectively address these issues [95,96]. In one report of the cross-linking strategy for polyimides, Xu et al. [97] developed cross-linked brominated 6FDA-based polyimide membranes by a thermal debromination process. This process eliminated the bromomethyl group to produce benzene radicals and subsequently formed covalent cross-links within the polymer network. This covalently cross-linked network structure significantly increased chain rigidity and limited the mobility of the segments, resulting in an excellent resistance to CO_2_-induced plasticization. Specifically, these cross-linked 6FDA-based polyimide membranes suffered from plasticization at a CO_2_ pressure of 41.4 bar, which was significantly higher than the 10.3 bar observed for the non-crosslinked membranes. In another study on the incorporation of nanofiller, Wang et al. [96] fabricated 6FDA-DAM polyimide-based MMMs incorporating ZIF-301 MOF for CO_2_/CH_4_ separation, which effectively integrated the advantages of fillers and polymers to significantly enhance separation performance. As shown in Figure 11A, when the MOF loading reached 20 wt%, the pure CO_2_ permeability was increased to 825 Barrer and the ideal selectivity of CO_2_/CH_4_ increased up to 24.9. The membrane showed a good plasticization resistance as the feed pressure increased from 0.5 MPa to 3.0 MPa. However, upon increasing the loading of ZIF-301 MOF crystals to 30 wt%, there was a decline in both the CO_2_ permeability and CO_2_/CH_4_ selectivity. This phenomenon was mainly attributed to the agglomeration of ZIF-301 fillers in the polymer matrix, which caused a decrease in gas separation performance.

In addition, the copolymerization strategy has also been applied to the separation of CO_2_/CH_4_. Fan et al. [98] synthesized fluorinated cardo-based polyimide membranes via the copolymerization of fluorinated-cardo-based diamine (FFDA) with 6FDA and DAM, achieving an exceptional gas separation performance that surpassed the 2008 Robeson upper bounds for CO_2_/CH_4_ (Figure 11B). Rigid cardo groups with C-F single bonds increased the packing efficiency of polymer chains, which endowed the 6FDA-FFDA/DAM (1:1) copolyimide membrane with a high CO_2_/CH_4_ separation performance beyond the 2008 CO_2_/CH_4_ Robeson upper bound. The ideal gas selectivity factor was 43.4, and the CO_2_ permeability was 203.4 Barrer. Based on copolymerization, this has been further modified by combining with thermal rearrangement strategies. Fan et al. [99] incorporated FFDA into the polybenzoxazole structure to synthesize a polyimide precursor. PI precursors were transformed into PBO structures using a thermal rearrangement strategy, which resulted in the formation of approximately 3 Å microporous structures, leading to an increase in gas diffusivity. Additionally, aromatic fluorine atoms in FFDA enhanced CO_2_ adsorption, thereby improving CO_2_/CH_4_ selectivity. Thermally rearranged 6FDA-FFDA/6FAP (1:1)-TR400 membranes exhibited a high separation performance, specifically, the permeability of CO_2_ reached 185.3 Barrer and the selectivity of CO_2_/CH_4_ reached 79.9. Compared with the 6FDA-FFDA/DAM (1:1) copolyimide membrane, the CO_2_/CH_4_ selectivity of the thermal rearranged 6FDA-FFDA/6FAP (1:1) membrane increased from 43.4 to 79.9, which was much higher than that of the 6FDA-FFDA/DAM (1:1) copolyimide membrane.

The CO_2_/CH_4_ separation (single gas) performance of polyimide membranes is comprehensively summarized in Table 1. To date, various modification techniques for polyimide membranes, such as copolymerization, blending, thermal rearrangement, and cross-linking, have been applied for CO_2_/CH_4_ separation. Among these methods, MMMs obtained by blending with nanofillers are particularly favored due to their competitive gas separation performance, along with mechanical and chemical stability. Furthermore, the compatibility between fillers and polymers remains a crucial factor in achieving efficient CO_2_/CH_4_ separation.

### 4.2. H_2_/CH_4_ Separation

Hydrogen-based energy, with its high energy density, low carbon emissions, and sustainability, is seen as a key component of the post-fossil fuel era. To utilize it effectively, producing high-purity hydrogen through efficient separation technologies is essential. Steam methane reforming currently represents the most prevalent method for hydrogen production worldwide. This reaction ideally generates a mixture dominated by hydrogen and carbon dioxide. However, due to reactor conversion efficiencies below 30%, unreacted methane accumulates in the process stream. It is important to separate H_2_/CH_4_ to achieve sufficient reagent conversion. By-products of industrial processes (such as dehydrogenation reactions and coking coal) can also produce H_2_ gas. Coke oven gas contains 54–59% H_2_, 21–28% CH_4_, and small amounts of other gases. The efficient separation of hydrogen from this mixture is essential to enable its effective utilization in downstream applications [106,107]. Although polyimide membranes were not used in industrial hydrogen separation until 1987, they have already gained significant attention for H_2_/CH_4_ separation [68].

**Figure 12:**
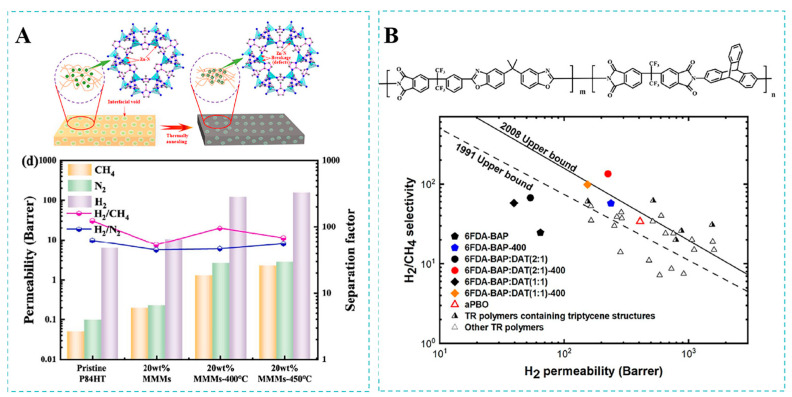
(**A**) Gas separation properties of MMMs with 20 wt% ZIF-8 loadings before and after thermal annealing. Reproduced with permission from ref. [109]. Copyright 2024, Elsevier B.V. (**B**) H_2_/CH_4_ separation performance of TR membranes and precursor membranes. Reproduced with permission from ref. [111]. Copyright 2025, Elsevier B.V.

To address the inherent compatibility of fillers and polymers, Wang et al. [108] developed an interfacial design strategy to enhance MMM gas separation performance, coating nanosized ZIF-8 with an ultrathin polydopamine layer. The polydopamine coating not only protected the pores of ZIF-8 zeolite from being blocked, but also acted as a compatibilizer to improve ZIF-8/polymer matrix interactions, thereby decreasing interface defects. The permeability of H_2_ showed a significant enhancement, which increased from 390 Barrer for pure PI membranes to 1858 Barrer for ZIF-8@PD-PI (30%) membranes. Compared with ZIF-8-PI (30%) membranes, the ideal selectivity of H_2_/CH_4_ was improved, which indicated that the polydopamine coating contributed to enhancing the selectivity of the MMMs. Furthermore, ZIF-8@PD-PI demonstrated an excellent long-term stability. This work introduced a novel design for high-performance MMMs, offering a potential strategy to overcome the existing interfacial compatibility issue between polymers and fillers. In their latest report, Mu et al. [109] proposed an innovative in situ defect engineering approach for ZIF-8 within mixed MMMs. The in-situ defect engineering of ZIF-8 was successfully achieved by the thermal treatment of MMMs at 400–450 °C. The process resulted in partial Zn-N bond breakage in ZIF-8, significantly expanding the BET specific surface area of ZIF-8 (from 1359 m^2^/g to 1790 m^2^/g). This effectively increased the adsorption sites and diffusion channels for gases, especially H_2_. Meanwhile, the polyimide matrix achieved a cross-linking reaction during heat treatment. This reaction repaired the existing interfacial defects between the ZIF-8 particles and the polymer matrix and improved the interfacial compatibility. At a 450 °C annealing temperature, the H_2_ permeability of MMMs with 20 wt% ZIF-8 reached 157.6 Barrer, which was improved by 23.6 times compared to the pristine membrane (Figure 12A).

Currently, many studies have proposed copolymerization strategies to improve the durability of membrane materials [110]. Huang et al. [110] designed polyimides with varying amounts of ether oxygen groups using a copolymerization strategy. The results showed that the tensile strength of 6FD/C (1:1)-SBI was 87.73 MPa and the elongation at break was 12.67%, which were significantly higher than those of 6FD/C (1:0)-SBI (4.61%). Copolymer tensile strength increased concomitantly with 6FCDA content, demonstrating a significant influence of dianhydride stoichiometry on mechanical properties. Specifically, ether linkages within the biphenyl moiety enhanced interactions between polymer backbone units, thereby improving intermolecular forces and overall mechanical performance. Additionally, they found that increasing the ether oxygen content significantly enhanced gas permeability. Both 6FD/C (1:1)-SBI and 6FD/C (1:3)-SBI membranes demonstrated a high H_2_ permeability, reaching the 2008 upper bound; however, their H_2_/CH_4_ selectivity still needed improvement. To enhance the H_2_/CH_4_ selectivity of copolyimide membranes, Wang et al. [111] synthesized a novel TR polymer through the polymerization of an amino-functionalized triptycene structure with a thermally rearrangeable o-hydroxyl group. Thermal conversion to polybenzoxazole during rearrangement prevented chain stacking and increased interchain distance, thereby improving H_2_ permeability. Moreover, the unique spatial arrangement of triptycene units facilitated molecular sieving. The H_2_ permeability of the 6FDA-BAP:DAT (2:1) membrane thermally rearranged at 400 °C was 225.3 Barrer, with a H_2_/CH_4_ selectivity of 146.3 (Figure 12B). The mechanical properties of the TR polymer were strengthened by π-π stacking interactions among triptycene units.

These findings suggest that the H_2_/CH_4_ separation performance of polyimide membranes can be effectively enhanced through blending nanofillers with polymers to create MMMs, copolymerization, and thermal rearrangement strategies, offering promising options for industrial applications. A comprehensive summary of polyimide membrane performance in H_2_/CH_4_ separation is provided in Table 2.

### 4.3. C_3_H_6_/C_3_H_8_ Separation

In a nature report, Sholl and Lively [116] reported that olefin/paraffin separation ranks among the seven transformative chemical separation technologies critical to global industrial advancement. Olefin/paraffin separation constitutes 0.3% of global energy consumption in propylene purification alone, exemplifying its status as an energy-intensive critical process. Driven by carbon neutrality goals, developing energy-efficient separation technologies is imperative. Notably, PI membranes demonstrate a superior propylene/propane separation performance among polymeric materials [74,117].

Nevertheless, under practical gas separation conditions, these membrane materials frequently exhibit low selectivity, significant plasticization effects, and inadequate long-term operational stability [118]. Specifically, research has demonstrated that polyimide is prone to plasticization in C_3_H_6_/C_3_H_8_ mixed gas even at low pressures, which significantly reduces separation selectivity [119]. Research efforts have shifted toward the design and modification of polymer structures via thermal annealing to enhance resistance to plasticization under actual gas operating conditions. For instance, Mita et al. [120] adopted thermal annealing at 210 °C for 18–20 h for 6FDA-6FpDA membranes. Thermal annealing induced rearrangement and close packing of the polymer chains, reducing the FFV. This structural change resulted in the 6FDA-6FpDA membranes exhibiting good anti-plasticization properties at a feed pressure of 4.8 bar [120]. In another study, Swaidan et al. [121] enhanced intermolecular interactions by thermally annealing the hydroxyl-containing polymer PIM-PI-OH to produce charge-transfer complexes (CTCs) within or between the chains of the molecule, which was helpful in suppressing inter-chain motions and promising for plasticization suppression. Upon the annealing of PIM-6FDA-OH at 250 °C, the selectivity of C_3_H_6_/C_3_H_8_ increased from 19 to 30.

However, polyimide membranes face an ongoing challenge in simultaneously enhancing their permeability and selectivity for emerging high-demand applications to broaden their applications. To address these issues, the incorporation of inorganic fillers into the polymeric matrix has received significant research attention on account of its potential to overcome the trade-off effect. Zhang et al. [122] fabricated MMMs by adding ZIF-8 to 6FDA-DAM, which enhanced C_3_H_6_ permeation compared to pure polyimide membranes. When the loading content of ZIF-8 reached 48%, the C_3_H_6_ permeability of the ZIF-8/6FDA-DAM MMMs was 56.2 Barrer, while the ideal selectivity of C_3_H_6_/C_3_H_8_ was 31.0, 258% and 150% higher than those of the pure 6FDA-DAM membrane, respectively, for permeability and selectivity. However, higher loadings of ZIF-8 caused the formation of defects that restricted direct industrial application. To improve the compatibility of ZIF-8 and 6FDA-DAM, Lee et al. [123] fabricated MMMs incorporating the amine-induced defect ZIF-8 (AZIF-8). The alkylamine groups on the surface of AZIF-8 were more compatible with the polymer matrix. Furthermore, MMMs with high loading 50% fillers showed a permeation flux of 79.38 Barrer for C_3_H_6_ and a separation coefficient of 39.8 for C_3_H_6_/C_3_H_8_.

**Figure 13:**
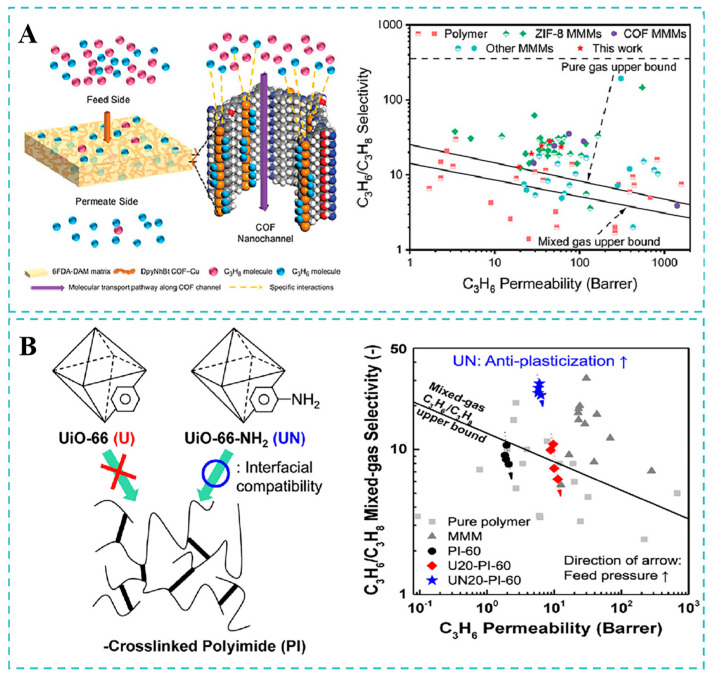
(**A**) C_3_H_6_/C_3_H_8_ separation performance of DpyNhBt COF-Cu/6FDA-DAM MMMs. Reproduced with permission from ref. [124]. Copyright 2023, Wiley-VCH. (**B**) C_3_H_6_/C_3_H_8_ separation performance of UiO-66-NH_2_/PI MMMs. Reproduced with permission from ref. [125]. Copyright 2022, Elsevier B.V.

In order to improve the separation performance of mixed matrix membranes, researchers have proposed the introduction of selective metal ions to develop facilitated transport membranes. Cheng et al. [124] developed novel DpyNhBt COF-Cu/6FDA-DAM MMMs incorporating Cu(I) covalent organic frameworks, where C_3_H_6_ interacted with Cu(I), thus promoting the transport of C_3_H_6_ (Figure 13A). The optional DpyNhBt COF-Cu/6FDA-DAM mixed matrix membrane showed a permeability of 49.3 for C_3_H_6_, respectively, in single gas permeation experiments, with a corresponding C_3_H_6_/C_3_H_8_ selectivity of 23.3. On the one hand, DpyNhBt COF-Cu strongly interacted with C_3_H_6_ rather than C_3_H_8_, attributed to the π-complexation effect between Cu^+^ and C_3_H_6_. On the other hand, these COF materials possessed a high specific surface area and regular pore structure, which enhanced gas permeability. In addition, the membranes still maintained an excellent separation performance after a 72 h stability test.

Lee et al. [125] investigated the synergistic effects of dual-modification strategies on membrane performance. They proposed two strategies (diamino cross-linking of polyimides and MOF functionalization strategy to enhance interfacial compatibility) to improve the separation performance of C_3_H_6_/C_3_H_8_ (Figure 13B). The surface amino groups of UiO-66-NH_2_ with the cross-linked polyimide formed strong hydrogen bonds, enhancing interfacial compatibility. Compared with pure polymer, the glass transition temperature of the UiO-66-NH_2_-incorporated MMM increased, which also explains the good interfacial compatibility between the filler and the polymer. When the filler concentration was 20 wt% and the cross-linking time was 60 s, the MMM demonstrated a C_3_H_6_/C_3_H_8_ selectivity of 23.7 under the harshest operation conditions. Moreover, the strong interaction between the functionalized filler and the cross-linked polymer matrix caused a remarkable anti-plasticization performance compared to both the pure polyimide membranes and the UiO-66/6FDA-DAM MMMs.

Table 3 summarizes the comprehensive performance of polyimide membranes in the separation of C_3_H_6_/C_3_H_8_. To date, various modification strategies for polyimide membranes have been applied to enhance C_3_H_6_/C_3_H_8_ separation. Among these, MMMs fabricated by incorporating nanoporous fillers (e.g., ZIF-8 and ZIF-67) exhibit exceptional promise due to their ability to simultaneously boost both permeability and selectivity. Critically, the good compatibility of filler and polymer remains paramount to suppress interfacial defects and plasticization under mixed gas conditions. In conclusion, to further develop novel polyimide membrane materials for C_3_H_6_/C_3_H_8_ separation, it is necessary not only to overcome the trade-off effect between permeability and selectivity, but also to address the plasticization phenomenon at a low pressure to be applicable for industry.

## 5. Conclusions and Outlook

PI, characterized by its high gas selectivity, exceptional thermal stability, chemical resistance, and mechanical strength, exhibits significant potential in the field of gas separation. We summarize the synthesis methods and membrane preparation approaches for PI and the strategies of membrane modification (thermal rearrangement, cross-linking, and physical blending). Research advances in PI for gas separation applications (such as CO_2_/CH_4_, H_2_/CH_4_, and C_3_H_6_/C_3_H_8_) are summarized. Moving forward, the advancement of innovative polyimide membrane materials will remain crucial in gas separation; nevertheless, several challenges demand further and comprehensive study.

(1) PI membranes are prone to plasticization when exposed to high-pressure gases such as CO_2_, leading to diminished selectivity. A series of novel polyimides with good anti-plasticization properties have been synthesized by monomer structure design, thermal rearrangement, and cross-linking modification of polyimides.
(2) As with most membrane materials, the persistent challenge for polyimide membranes is to balance selectivity and permeability. Achieving simultaneous enhancement in both properties remains a core goal in the development of high-performance gas separation membranes. Preparation of MMMs by incorporating nanofillers into the PI matrix can improve the separation performance of membranes. However, the compatibility between polymer matrices and inorganic fillers in MMMs remains a critical challenge. This issue can be addressed through the chemical structure modification of fillers or polymers to reduce or eliminate interfacial defects.
(3) Designing high-performance PI membranes is still difficult, often relying on time-consuming empirical trial-and-error experiments. Machine learning accelerates the discovery of high-performance PI materials through the effective screening of extensive databases using active learning and multi-objective methods. This approach enhances the precision of property predictions, reduces reliance on experimental data, and provides guidance for the design of molecular structures.

In summary, PI membranes represent a highly attractive option for gas separation applications. Although challenges remain, ongoing advancements in material design, synthesis methods, and modification strategies are expected to significantly improve their performance and broaden their industrial applications. As research progresses, polyimide-based membranes are likely to play an increasingly vital role in addressing the energy and environmental challenges associated with gas separations.

## Figures and Tables

**Table 1:** Examples of polyimide-based membranes for CO_2_/CH_4_ separation.

Membrane	Temperature (°C)	Pressure (Bar)	Permeability (Barrer)	Selectivity	Ref.
P84	35	10.1	1.2	50	[100]
Matrimide^®^ 5218	35	2	8.3	31	[87]
6FDA-DABA	35	4.5	10.9	34.1	[85]
6FBDA	35	4.6	14.0	48.3	[85]
50%ZIF-8-NH2/6FBDA	35	4.7	80.8	89.4	[85]
6FDA-FFDA/DAM (1:1)	35	2.0	203.4	43.4	[98]
6FDA-6FAP/TAPA (7:3)	25	3.5	26.9	134.5	[101]
6FDA-APAF-350	35	6.9	10.5	74.0	[102]
6FDA-APAF/DABA-350	35	6.9	26.6	60.0	[102]
20%UiO-66/6FDA-SDA	35	10.0	32.7	40.0	[103]
19%UiO-66-NH_2_/6FDA-DAM	35	3.0	1385.6	20.25	[104]
10%ZIF-8/6FDA-DAM	25	2.0	1689.0	16.6	[105]

**Table 2:** Examples of polyimide-based membranes for H_2_/CH_4_ separation.

Membrane	Temperature (°C)	Pressure (Bar)	Permeability (Barrer)	Selectivity	Ref.
P84 HT/20% ZIF-8(400 °C)	35	2.0	123.8	75	[109]
Matrimide^®^ 5218/15% DDR	35	10.0	29.37	309.16	[112]
Matrimide^®^ 5218/20% DDR	35	10.0	34.9	375.27	[112]
30%ZIF-8/6FDA-BI	35	4.0	174	140.9	[113]
6FDA-DAM:DABA (3:2)/10%ZIF-8-90(30)	35	4.0	301	55.7	[114]
20%-COOH-PI/NH_2_-UiO-66	25	4.0	1180	27.2	[115]
50%ZIF-8-NH_2_/6FBDA	35	4.5	127.5	141.7	[85]
6FDA-BAP	35	2.0	64.57	24.6	[111]
6FDA-BAP:DAT (2:1)	35	2.0	53.61	67.6	[111]
6FDA-BAP:DAT (2:1) 400 °C	35	2.0	225.29	146.3	[111]

**Table 3:** Examples of polyimide-based membranes for C_3_H_6_/C_3_H_8_ separation.

Membrane	Temperature (°C)	Pressure (Bar)	Permeability (Barrer)	Selectivity	Ref.
48%ZIF-8/6FDA-DAM	35	2.0	56.2	31	[122]
20%ZIF-8/GO-6FDA-DAM	35	2.0	43.1	13.9	[126]
PIM-6FDA-OH	35	2.0	3.5	30	[88]
65%ZIF-8/PIM-6FDA-OH	35	2.0	38	43	[88]
40%ZIF-67@Ag_4_tz_4_/6FDA-TMPDA	30	2.0	127	23.3	[127]
6FDA-TMPDA	30	2.0	78	9.5	[127]
30%ZIF-67/6FDA-TMPDA	30	2.0	157.6	14	[127]
6FDA-DAM	35	2.0	33.29	10.28	[78]
Br-6FDA-DAM	35	2.0	7.27	14.04	[78]
Br-6FDA-DAM@380 °C	35	2.0	84.7	14.6	[78]
15%CN-ZIF-8/6FDA-DAM	35	3.0	379.8	23.6	[128]

## Data Availability

Data will be made available on request.

## Abbreviations

The following abbreviations are used in this manuscript:

PSf	Polysulfone
CA	Cellulose acetate
PI	Polyimide
*T*_g_	Glass transition temperature
MMMs	Mixed matrix membranes
PMDA	Pyromellitic dianhydride
6FDA	4,4′-(hexafluoroisopropylidene)-diphthalic anhydride
ODPA	3,3′,4,4′-oxydiphthalic anhydride
BPDA	3,3′,4,4′-biphenyltetracarboxylic dianhydride
BTDA	3,3′,4,4′-benzophenonetetracarboxylic dianhydride
DAM	2,4,6-trimethyl-m-phenylenediamine
ODA	4,4′-oxydianiline
FDA	9,9-bis(4-aminophenyl) fluorene
Durene	2,3,5,6-tetramethyl-1,4-phenylenediamine
PAA	Polyamide acid
DMF	N, N-dimethylformamide
NMP	1-methyl-2-pyrrolidone
GVL	Gamma-Valerolactone
TR	Thermally rearranged
PBO	Polybenzoxazole
TB	Tröger’s Base
UV	Ultraviolet
FFV	Fractional free volume
FFDA	Fluorinated-cardo-based diamine
CTCs	Charge-transfer complexes
MOF	Metal–Organic Framework
COF	Covalent Organic Framework
